# Injecting postpartal gilts or sows with a supraphysiological dose of oxytocin: effects on sow and piglet performances

**DOI:** 10.1093/tas/txae091

**Published:** 2024-06-12

**Authors:** Chantal Farmer, Sylvie-Anne Bolduc, Frédérick Guay, Isabelle Cormier

**Affiliations:** Agriculture and Agri-Food Canada, Sherbrooke Research and Development Centre, 2000 College, Sherbrooke, QC, Canada J1M 0C8; Department of Animal Science, Laval University, Québec, QC, Canada G1V 0A6; Department of Animal Science, Laval University, Québec, QC, Canada G1V 0A6; Sollio Agriculture, Frampton, QC, Canada G0R 1M0

**Keywords:** mortality, oxytocin, parturition, piglet performance, postpartum, sow

## Abstract

The goal of this project was to determine the effects of a supraphysiological dose of oxytocin given to gilts or multiparous sows 8 to 12 h after the end of farrowing on the performance of their progeny. Sows from three parity groups (1 = parity 1; 2 = parities 2, 3, and 4; 3 = parities 5 to 14) received no injection (CTL, controls; *n* = 17, 27, and 23 for parity groups 1, 2, and 3, respectively) or one intramuscular injection of 75 IU of oxytocin (OXY, *n* = 17, 24, and 26 for parity groups 1, 2, and 3, respectively) 8 to 12 h after birth of the last piglet. Colostrum samples were obtained 8 h after oxytocin injection in 18 sows from parity group 2 (CTL, *n* = 10; OXY, *n* = 8). Standard milk composition was measured as well as the Na/K ratio, and IGF-1, IgG, and IgA concentrations. The same sows were used to obtain blood samples from four male piglets of average litter body weight (BW) 8 h post-treatment to measure concentrations of IGF-1, IgG, and IgA. Piglets and sows were weighed at farrowing and weaning (day 21) and sow feed intake and piglet mortality were recorded. There was no effect of OXY on sow or piglet BW at any measured times and percent preweaning piglet mortality was not affected by OXY or parity. First-parity sows had lower BW than multiparous sows at both times (*P *< 0.001), and piglet average daily gain from birth to weaning was greater in parity group 2 compared with first-parity litters (*P *< 0.05). Average daily sow feed intake over the first week of lactation tended to be greater in OXY vs CTL sows (*P* = 0.07), and multiparous sows consumed more feed than first-parity sows on all weeks of lactation (*P *< 0.001). Eight hours after treatment, there was a tendency for colostral Na to be greater in OXY vs CTL sows (*P *= 0.06), and none of the measured variables in piglet blood were affected by treatment. In conclusion, injecting 75 IU of oxytocin 8 to 12 h after the birth of the last piglet did not prolong the period of colostrogenesis or improve the growth or survival of piglets and this was consistent across parities.

## Teaser

Injecting 75 IU of oxytocin 8 to 12 h after birth of the last piglet did not prolong the period of colostrogenesis or improve the growth or survival of piglets from primiparous or multiparous sows.

## Introduction

The problem of high morbidity and mortality of neonatal piglets is still widespread and has been worsened with the current use of hyperprolific sow lines. Even though many attempts were made in the past years to develop novel strategies during gestation, the transition period or early lactation in order to improve piglet survival (review by Farmer and Edwards, 2022), 15% to 20% of all piglets born still die either during the farrowing process or in early lactation (review by Baxter and Edwards, 2018). Colostrum is essential to provide energy for thermoregulation and passive immunity to neonatal piglets, and the ingestion of a minimum amount of 250 g of colostrum is key to ensure piglet survival (Quesnel et al., 2012). Many physiological traits of piglets, such as low birth weight and poor vitality, render them most susceptible to consume inadequate colostrum (Quesnel et al., 2023). Furthermore, colostrum yield does not increase with litter size so the amount of colostrum ingested per piglet in large litters is lower compared with smaller litters (Devillers et al., 2007; Vadmand et al., 2015). In fact, Quesnel et al. (2012) reported that most sows do not produce enough colostrum for all piglets in a litter to obtain the critical 250 g. Therefore, any attempt at increasing colostrum yield would be beneficial for suckling piglets.

The status of the tight junctions between mammary epithelial cells affects colostrogenesis, with more open junctions allowing for the passage of larger molecules from the bloodstream to the colostrum (Nguyen and Neville, 1998). The leakiness of mammary tight junctions decreases as lactation advances and this timing can be altered with exogenous oxytocin. The period of colostrogenesis was prolonged by injecting one supraphysiological dose of oxytocin to sows in the early postpartum period (Farmer et al., 2017). When 75 IU of oxytocin was injected 12 to 20 h after birth of the last piglet, the Na/K ratio in milk (indicative of leakiness of tight junctions) was increased 8 h post-injection. The composition of early milk was also improved with concentrations of insulin-like growth factor-1 (**IGF-1**), protein, energy, and immunoglobulin A (**IgA**) being greater in treated versus control sows. Taking into account that the quality of colostrum decreases very rapidly after farrowing (Theil et al., 2014), it may be that injecting such a high dose of oxytocin earlier postpartum could be more advantageous by maintaining higher levels of the various colostral components over a period of time.

It is well-established that piglets from primiparous sows have lower birthweights and lower weaning weights than piglets from multiparous sows (Miller et al., 2012; Craig et al., 2017). Furthermore, providing supplemental milk to nursing piglets improved the growth rate of gilt progeny but not sow progeny (Miller et al., 2012). Recent results also showed that piglets born from gilts have reduced gastrointestinal tract development compared with progeny from multiparous sows, which could partly account for their poorer growth performance (Craig et al., 2019). It has been suggested that management strategies aiming at increasing sow milk yield would be most advantageous for gilt progeny compared with sow progeny (Wijesiriwardana et al., 2024). Hence, the goal of the present study was to determine the effects of a supraphysiological dose of oxytocin given to primiparous or multiparous sows 8 to 12 h after the end of farrowing on the performance of their progeny.

## Materials and Methods

Animals were cared for according to a recommended code of practice (CCAC, 2009) following procedures approved by the institutional animal care committee of the Sherbrooke Research and Development Center of Agriculture and Agri-Food Canada. The experiment was carried out at the Research Farm of Sollio, Agriculture in Frampton, QC.

### Animals and Treatments

One hundred and thirty-four sows (Yorkshire × Landrace, F1, AlphaGene, Saint-Hyacinthe, QC, Canada over three parity groups (group 1 = parity 1; group 2 = parities 2, 3 and 4; group 3 = parities 5 to 14) were divided into the following two treatment groups: 1) receiving no injection (CTL, negative controls; *n* = 17, 27, and 23 for parity groups 1, 2, and 3, respectively) or 2) receiving one intramuscular injection of 75 IU (3.75 mL) of oxytocin (Vetoquinol, Lavaltrie, QC, Canada) in the neck (OXY, *n* = 17, 24, and 26 for parity groups 1, 2, and 3, respectively) between 8 and 12 h after birth of the last piglet. Sows were bred using pools of semen from Duroc Porc Coop boars (Center d’Insémination Porcine du Québec, Saint-Lambert-de-Lauzon, QC, Canada) and kept in individual crate (0.8 × 2.0 m) for all gestation. Five days before expected farrowing, sows were transferred to the farrowing crate (1.5 × 2.4 m) and no farrowing induction was performed. Sows were not given any oxytocin during parturition. Litters were standardized to 12 ± 2 piglets within 24 h of birth by cross-fostering within treatment. Piglets were weighed individually at birth, on day 1 (after cross-fostering), and at weaning (day 21) and received no creep feed. Preweaning mortalities were recorded and sow health was observed. There were no instances of mastitis or other disease.

During gestation, sows were fed one daily meal (0800 hours) of a conventional corn-based diet that contained 13.0% CP, 3,100 kcal/kg digestible energy, 0.60 lysine, 0.72% calcium, and 0.61% phosphorus. Sows were fed according to their body condition at mating and they received an average of 2.7 kg/d until day 96 at which time they received 3.0 kg/d until farrowing. Around day 110 of gestation, sows were fed 3.18 kg daily of a commercial corn–soya diet containing 17.1% CP, 3,445 kcal/kg digestible energy, 1.09% lysine, 0.82% calcium, and 0.56% phosphorus. On the day of farrowing (day 0), sows received two portions of 1.59 kg of this diet, followed by 4.08 kg/d on day 1, 5.90 kg on day 2, 7.71 kg on day 3, and ad libitum thereafter until weaning. Refusals were weighed daily to obtain feed intakes. Sows were weighed at mating, on day 110 of gestation, after farrowing (12 to 24 h post-farrowing), and at weaning (day 20 ± 2 of lactation). Sow backfat thicknesses (**BF**) were measured ultrasonically at P2 of the last rib (WED-3000, Shenzhen WELLD Medical Electronics Co., Ltd., Schenzhen, China) at the same time sows were weighed, except for after farrowing.

Eighteen litters from sows in parity group 2 (CTL, *n* = 10, average parity 3.10 ± 0.88; OXY, *n* = 8, average parity 2.83 ± 0.75) were randomly selected for piglet blood sampling. Blood was collected by jugular venipuncture from four male piglets of average litter body weight (BW) 17 ± 1 h after birth of the last piglet, exactly 8 h after the injection of oxytocin in the case of OXY sows. The same 18 sows were used to obtain samples of colostrum between 0 and 4 h after the onset of parturition and 8 h after the injection of oxytocin, for OXY sows, or 17 ± 1 h after birth of the last piglet for CTL sows.

### Blood Handling and Assays

The concentrations of IGF-1, immunoglobulin G (**IgG**), and IgA were measured in piglet blood samples (10 mL) that were collected in EDTA tubes (Becton Dickinson, Franklin Lakes, NJ), put on ice and centrifuged within 20 min for 12 min at 1,800 × *g* at 4 °C. Plasma was then immediately recovered and stored at −20 °C. Concentrations of IGF-1 were measured with a commercial RIA kit for human IGF-1 (ALPCO Diagnostics, Salem, NH) with minor modifications as detailed previously (Plante et al., 2011). The assay was validated using a pooled plasma sample from piglets, as was previously described (Plante et al., 2011). Sensitivity of the assay was 0.10 ng/mL, while the intra- and interassay CVs were 5.48% and 6.14%, respectively. Concentrations of IgG, and IgA were measured by ELISA. The following commercial kits were used in accordance with manufacturer recommendations after proper validation: porcine IgG (Bethyl Laboratories Inc., Montgomery, TX, USA), and porcine IgA (Bethyl Laboratories Inc.). Intra- and interassay CV were, respectively, 2.97% and 9.10% for IgG, and 5.80% and 1,06% for IgA.

### Colostrum Handling and Analyses

Samples of colostrum were obtained by hand milking as many glands as possible (without using oxytocin) and then pooling samples (20 mL total) for each sow. Upon collection colostrum was rapidly refrigerated, then mixed for 1 min with an agitator and filtered through two layers of gauze before being mixed again for 10 min. Homogenized samples were aliquoted and stored at −20 °C to be later analyzed for contents of dry matter (Method 950.46; AOAC, 2005), fat (Method 991.36, AOAC, 2005), protein (Method 928.08; AOAC, 2005), lactose, sodium, potassium, IgG, and IgA. Lactose was measured with an enzymatic method using a commercial kit (Neogen, Lansing, MI, USA) with 30 µL of galactosidase and a final incubation time of 60 min. The concentrations of Na and K were measured with an atomic absorption spectrometer (PerkinElmer AAnalyst 300, Normalk, CT). Concentrations of IgG and IgA were determined with commercial ELISA kits (Bethyl Laboratories Inc., Montgomery, TX, USA). All above colostrum analyses were properly validated and showed intra- and interassay CVs below 3.0%, except for IgG and IgA which were below 7.5%.

Concentrations of IGF-I were measured in lactoserum obtained by centrifuging thawed fresh milk twice for 60 min at 51,500 × *g* at 4°C and harvesting the middle phase. The same method as described for plasma was used in lactoserum. The commercial ELISA (ALPCO Diagnostics, Salem, NH) kit was validated and showed parallelism of 101.08% and an average mass recovery of 96.76%. The intra- and interassay CV were 2.39% and 4.62%, respectively.

### Statistical Analyses

The MIXED procedure of SAS (SAS Inst. Inc., Cary, NC) was used for statistical analyses. The model for all zootechnical variables measured included the effects of treatment (oxytocin), parity group, and their interaction. The model for colostrum composition and blood variables in piglets included only the effect of treatment (oxytocin). Repeated measures ANOVAs with the main factors treatment (the error term being sow within treatment) and time of sampling (the residual error being the error term), and the treatment by time interaction, were also performed on colostrum composition data, with separate analyses of variance for each sampling time also being carried out. An ANOVA using the GLIMMIX procedure with a beta distribution was used to assess treatment and parity effects on two values of percent preweaning mortality, namely, from birth until weaning and also from after cross-fostering until weaning. Data in Tables are presented as least squares means ± maximal SEM. Results are considered not significant when *P* > 0.10.

## Results

BW, BF, and lactation feed intake of sows are shown in Table 1. There was no effect of oxytocin treatment on BW or BF at any of the measured times, and neither BW or BF losses during lactation were affected by OXY. On the other hand, parity affected BW at all measured times with first-parity sows having lower BW than multiparous sows (*P *< 0.001), and the pairwise comparison also showed a greater lactation BW loss for primiparous sows (*P* < 0.05). Parity affected BF on day 20 of lactation only, with sows from parity group 2 having less BF than those from parity group 3 (*P *< 0.05), and parity group 1 sows being intermediate. Even though sow BF loss during lactation was numerically lower (more than 10% difference) for parity group 3 compared with parity groups 1 and 2, this was not significant. Average daily feed intake over the first week of lactation tended to be greater in OXY versus CTL sows (*P* = 0.07), but this difference was no longer significant over weeks two or three. Multiparous sows (parity groups 2 and 3) consumed more feed than first-parity sows on all weeks of lactation (*P *< 0.001), with no significant treatment × parity interaction.

**Table 1.** Body weight, backfat thickness, and lactation feed intake of sows that received one intramuscular injection of 75 IU of oxytocin (OXY, *n* = 67), or no injection (CTL, *n* = 67), 8 to 12 h after the birth of the last piglet

	Treatment (T)		Parity group (P)			SEM^*^		*P-*value^†^	
Variable	CTL	OXY	1	2	3	T	P	T	P
*BW, kg*									
Mating	210.9	209.0	151.1^a^	217.6^b^	261.2^c^	2.8	3.8	0.636	0.001
Day 110 of gestation	266.4	261.6	227.2^a^	268.3^b^	296.6^c^	2.1	2.9	0.119	0.001
12 to 24 h postpartum	252.2	247.5	212.0^a^	253.3^b^	284.3^c^	2.2	3.0	0.125	0.001
Day 20 ± 2 of lactation	242.3	239.8	200.5^a^	245.6^b^	277.0^c^	2.6	3.5	0.495	0.001
Lactation BW loss	9.7	7.1	11.5^a^	6.9^b^	6.9^b^	1.3	1.8	0.157	0.091
*Backfat thickness, mm*									
Mating	16.6	16.5	15.9	16.1	17.6	0.5	0.7	0.845	0.087
Day 110 of gestation	18.8	18.6	18.6	18.0	19.5	0.5	0.7	0.683	0.171
Day 20 ± 2 of lactation	16.3	16.0	15.8^a,b^	15.5^a^	17.2^b^	0.5	0.6	0.699	0.052
Lactation backfat loss	2.6	2.6	2.9	2.6	2.3	0.3	0.4	0.937	0.459
*Mean feed intake, kg/d*									
Lactation week 1	4.92^d^	5.29^e^	4.50^a^	5.43^b^	5.40^b^	0.15	0.20	0.072	0.001
Lactation week 2	7.11	7.44	6.18^a^	7.96^b^	7.69^b^	0.15	0.20	0.125	0.001
Lactation week 3	8.01	8.12	7.02^a^	8.77^b^	8.39^b^	0.15	0.21	0.599	0.001

^*^Maximum value for the standard error of the mean (SEM).

^†^No significant T × P interaction for any variable (*P* > 0.10).

^a,b,c^Means within a row with different superscripts differ (*P *< 0.05).

^d,e^Means within a row with different superscripts tend to differ (*P *< 0.10).

Sows were from three parity groups, namely, group 1) parity 1 (*n* = 34), group 2) parities 2, 3 and 4 (*n* = 51), and group 3) parities 5 to 14 (*n* = 49).

Individual piglet BW, piglet average daily BW gain, total litter BW, and litter sizes are shown in Table 2. None of these measured variables were affected by the oxytocin treatment, whereas parity group did have effects, and there was no OXY × parity group interaction on any variable. Pairwise comparisons showed a tendency for greater BW at birth and at weaning for piglets from sows in parity group 2 compared to primiparous sows (*P* < 0.10). The piglet BW gain, whether expressed as total kilograms from birth to weaning or kg/d over that same period, was also greater in litters from parity group 2 compared with first-parity litters (*P *< 0.05). Total litter BW at birth and on day 1 after cross-fostering were greater for parity group 2 compared with groups 1 and 3 (*P* < 0.05), and this difference was still apparent at weaning with total litter BW being greatest for parity group 2 and differing significantly from parity group 3 (*P* < 0.05), with group 1 being intermediate. Litter size at birth was greatest in parity group 2 compared with group 3, and after cross-fostering it was greatest in both parity groups 1 and 2 compared with parity group 3 (*P *< 0.05). Litter size was still greatest in parity group 1 compared with parity group 3 at weaning (*P* < 0.05), with parity group 2 being intermediate. The percent preweaning piglet mortality (back-transformed data), whether considered from birth until weaning (14.6% and 16.6% for CTL and OXY sows, respectively, with lower and upper confidence intervals of 12.5% and 17.0% for CTL, and 14.5% and 18.9% for OXY) or from cross-fostering until weaning (11.8% and 11.8% for CTL and OXY sows, respectively, with lower and upper confidence intervals of 10.0% and 14.0% for CTL, and 10.1% and 13.7% for OXY) was not affected by treatment or by parity.

**Table 2.** Individual piglet BW, piglet BW gain, total litter BW, and litter sizes for sows receiving one intramuscular injection of 0.75 IU of oxytocin (OXY, *n* = 67), or no injection (CTL, *n* = 67), 8 to 12 h after birth of the last piglet

	Treatment (T)		Parity group (P)			SEM^*^		*P-*value^†^	
Variable	CTL	OXY	1	2	3	T	P	T	P
*BW, kg*									
Birth	1.40	1.37	1.36^c^	1.43^d^	1.37^d^	0.02	0.03	0.277	0.119
Day 1 (after fostering)	1.42	1.39	1.38	1.44	1.39	0.02	0.03	0.496	0.274
Day 21 (weaning)	6.44	6.34	6.21^c^	6.58^d^	6.38^c,d^	0.12	0.16	0.531	0.187
*BW gain,*									
Birth to weaning, kg	5.07	4.95	4.83^a^	5.22^b^	4.98^a,b^	0.11	0.15	0.413	0.115
Birth to weaning, kg/d	0.251	0.244	0.238^a^	0.257^b^	0.248^a,b^	0.005	0.006	0.232	0.064
*Total litter BW, kg*									
Birth	18.55	19.45	18.24^a^	21.09^b^	17.66^a^	0.54	0.74	0.237	0.001
Day 1 (after fostering)	18.32	18.24	18.12^a^	19.31^b^	17.41^a^	0.34	0.47	0.867	0.003
Day 21 (weaning)	77.45	76.94	77.40^a,b^	80.14^a^	74.04^b^	1.84	2.48	0.841	0.121
*Litter size*									
Total born alive	13.38	14.43	13.65^a,b^	14.88^a^	13.19^b^	0.42	0.57	0.076	0.037
Total after fostering	12.96	13.10	13.15^a^	13.43^a^	12.52^b^	0.14	0.19	0.486	0.004
Total at weaning	12.03	12.21	12.52^a^	12.19^a,b^	11.66^b^	0.20	0.27	0.528	0.049

^*^Maximum value for the standard error of the mean (SEM).

^†^No significant T × P interaction for any variable (*P* ≥ 0.10).

^a,b^Means within a row with different superscripts differ (*P *≤ 0.05).

^c,d^Means within a row with different superscripts tend to differ (*P *< 0.10).

Sows were from three parity groups, namely, group 1) parity 1 (*n* = 34), group 2) parities 2, 3, and 4 (*n* = 51), and group 3) parities 5 to 14 (*n* = 49).

The composition of colostrum 0 to 4 h after the onset of parturition and 17 ± 1 h after the birth of the last piglet (i.e., 8 h post oxytocin injection in OXY sows) in sows from parity group 2 is shown in Table 3. The concentration of IgA at 0 to 4 h after the onset of parturition differed between the two groups of sows, with values being greater in OXY compared with CTL sows (*P *< 0.05). However, this effect was no longer present at 17 h postpartum. On the contrary, 17 h after the end of farrowing, there was a tendency for colostral Na to be greater in OXY versus CTL sows (*P *= 0.06), whereas there was no treatment effect at 0 to 4 h postpartum.

**Table 3.** Composition of colostrum in sows receiving one intramuscular injection of 0.75 IU of oxytocin (OXY, *n* = 8), or no injection (CTL, *n* = 10), 8 to 12 h after birth of the last piglet

	Treatment			
Item	CTL	OXY	SEM^*^	*P*-value
*Dry matter, %* ^†^				
0 to 4 h postpartum	26.18	26.06	1.00	0.927
17 h postpartum	20.69	19.41	0.80	0.254
*Fat, %* ^†^				
0 to 4 h postpartum	5.73	5.73	0.44	0.998
17 h postpartum	6.87	6.91	0.58	0.960
*Protein, %* ^†^				
0 to 4 h postpartum	16.07	15.68	0.86	0.741
17 h postpartum	8.75	7.55	0.62	0.171
*Lactose, %* ^†^				
0 to 4 h postpartum	2.60	2.65	0.12	0.744
17 h postpartum	3.50	3.51	0.13	0.965
*Na, mg/L* ^‡^				
0 to 4 h postpartum	837.4	833.4	41.7	0.944
17 h postpartum	615.0	775.2	58.1	0.057
*K, mg/L* ^‡^				
0 to 4 h postpartum	1401.1	1497.2	50.5	0.175
17 h postpartum	1419.8	1345.1	62.2	0.384
*Na/K* ^‡^				
0 to 4 h postpartum	0.61	0.57	0.04	0.485
17 h postpartum	0.45	0.60	0.07	0.115
*IGF-I, ng/mL* ^†^ ^,^ ^∥^				
0 to 4 h postpartum	285.5	340.5	29.0	0.176
17 h postpartum	113.5	105.6	16.6	0.735
*IgG, mg/mL* ^†^ ^,^ ^∥^				
0 to 4 h postpartum	69.73	83.18	8.75	0.269
17 h postpartum	29.94	22.19	4.30	0.198
*IgA, mg/mL* ^‡^				
0 to 4 h postpartum	8.83	11.93	1.04	0.041
17 h postpartum	4.32	4.21	0.67	0.901

^*^Maximum value for the standard error of the mean (SEM).

^†^Time of sampling effect (*P* < 0.01).

^‡^Treatment × time interaction (*P* ≤ 0.05).

^∥^Tendency for treatment × time interaction (*P* < 0.10).

Colostrum samples were obtained 0 to 4 h after the onset of parturition and 17 ± 1 h after the birth of the last piglet from sows in parity group 2 (parities 2, 3, and 4).

Circulating concentrations of IgG (38.50 vs. 37.02 ± 3.2 mg/mL for CTL and OXY litters, respectively), IgA (8.54 vs. 9.59 ± 1.25 mg/mL for CTL and OXY litters, respectively), and IGF-1 (36.5 vs. 32.0 ± 2.9 ng/mL for CTL and OXY litters, respectively) in the blood of piglets from sows in parity group 2 at 17 ± 1 h after the end of farrowing were not affected by oxytocin treatment (*P *> 0.10).

## Discussion

Current findings provide important information on the potential use of a single injection of a supraphysiological dose of oxytocin in postpartal sows to improve the performance of their piglets. An earlier study (Farmer et al., 2017) showed greater quality of lacteal secretions 8 h after sows received 75 IU of oxytocin 12 to 20 h after birth of their last piglet. Such an improvement was not observed in the present study when the oxytocin was injected earlier (i.e., 8 to 12 h after birth of the last piglet). The timing of colostrum or milk collection relative to the oxytocin injection (8 h post-injection) was similar in both studies, but the greater concentrations of protein, Na, IGF-1, IgG, and IgA in lacteal secretions after treatment in the 2017 study were not reproduced in the current study. This difference is likely due to the fact that lacteal secretions in that previous study represented transient milk instead of colostrum (Theil et al., 2014). The only nutrient affected by post-oxytocin treatment in the current study was Na, and values for the Na/K ratio were much greater in the current than in the previous study, reflecting a larger space between mammary epithelial cells that is characteristic of the colostral period. Hence, findings suggest that timing of the oxytocin injection is important for its effect on composition of lacteal secretions. When the supraphysiological dose of oxytocin is injected when colostrum is still produced (as evidenced by high concentrations of protein, IgG, IgA, IGF-1, and high Na/K ratio in the current study), it does not appear to lead to a prolongation of the colostral phase, contrary to what was reported with a later injection (Farmer et al., 2017). It is most likely that all changes in the composition of lacteal secretions in the 2017 study were linked to the significant increase in the Na/K ratio allowing for increased passage of large molecules into transient milk (Nguyen and Neville, 1998). In the case of colostrum, such an increase in Na/K ratio could not be achieved, likely because it was already high, leading to an absence of treatment effect on other milk constituents.

The significant time × treatment interactions for colostral Na and Na/K ratio are due to similar values for the variables across treatments at 0 to 4 h postpartum, but increased values in OXY vs. CTL sows at 17 h postpartum. This finding illustrates looser tight junctions that should allow more passage of large molecules from the bloodstream to the colostrum in OXY sows. However, even though a 33% increase in Na/K ratio was present in OXY versus CTL sows at 17 h postpartum, this was not significant. Furthermore, the greater IgG concentrations in colostrum of OXY sows versus CTL sows at 0 to 4 h postpartum (before the oxytocin treatment) were not maintained until 17 h postpartum. Together, these results demonstrate that the timing of oxytocin injection in the current study had a lesser impact compared to that of the previous study (Farmer et al., 2017). The absence of OXY effect on composition of lacteal secretions was corroborated by the unaltered concentrations of the blood variables measured in suckling piglets. Even though colostral composition and blood variables in piglets were only measured in parity group 2, the absence of treatment effect or treatment × parity group interaction on BW gain or preweaning mortality of piglets suggests that colostral and blood results from parity group 2 are representative of what happened in parity groups 1 and 3. However, taking into account previous reports of greater immune status in older sows (Klobasa and Butler, 1987), it was expected that effects may have been greater in multiparous compared with primiparous sows. Yet, it is not known if parity would affect the positive response previously reported to a later injection of oxytocin.

Piglet growth was not affected by the oxytocin treatment in the previous study (Farmer et al., 2017), but only 10 sows per treatment were used, which was not enough to detect potential differences in this highly variable measure. The current study was carried out on a much larger number of sows in a commercial setting and the fact that piglet growth was not affected is either due to the different timing of oxytocin injection relative to the previous study, or to an absence of treatment effect. The former is most likely the case because the composition of lacteal secretions was not altered. However, it would be of interest to determine the impact of oxytocin given 12 to 20 h after the birth of the last piglet on the growth and survival of piglets in a larger-scale study.

## Abbreviations

BF: backfat thickness
BW: body weight
CTL: control
DM: dry matter
IGF-1: insulin-like growth factor-1
IgA: immunoglobulin A
IgG: immunoglobulin G
